# PTEN–Foxo1 signaling triggers HMGB1-mediated innate immune responses in acute lung injury

**DOI:** 10.1007/s12026-015-8639-z

**Published:** 2015-03-11

**Authors:** Min Zhou, Yadi Zhang, Xulin Chen, Jianjun Zhu, Min Du, Liang Zhou, Ling Zhang, Wei Wang, Gengyun Sun

**Affiliations:** 1 Department of Respiratory Medicine, The First Affiliated Hospital of Anhui Medical University, JiXi Road 218, Hefei, 230022 Anhui People’s Republic of China; 2 Department of Critical Care Medicine, The First Affiliated Hospital of Anhui Medical University, Hefei, Anhui People’s Republic of China; 3 Department of Burns, The First Affiliated Hospital of Anhui Medical University, Hefei, Anhui People’s Republic of China; 4 Department of Liver Surgery, Renji Hospital, Shanghai Jiaotong University School of Medicine, Shanghai, People’s Republic of China

**Keywords:** Macrophages, β-Catenin, Akt, TLR4, Lung inflammation

## Abstract

PTEN is a multifunctional phosphatase that regulates immune responses through a PI3K/Akt signaling cascade. HMGB1 plays an important role in the initiation of innate immune responses to induce acute lung injury (ALI). This study was designed to investigate the role of PTEN/Foxo1 signaling in the regulation of in vivo and in vitro innate immune responses in ALI. Using a mouse model of ALI, wild-type (WT) and myeloid-specific PTEN knockout (PTEN^M-KO^) mice were instilled with a recombinant HMGB1 (rHMGB1) or PBS. In some experiments, Foxo1 siRNA or non-specific siRNA was injected into mice 6 h prior to rHMGB1 instillation into lung. We found that rHMGB1 treatment in WT mice increased the expression of PTEN, Foxo1, TLR4, and NF-κB in alveolar macrophages from WT mice. However, macrophage-specific PTEN ablation resulted in reduced Foxo1 and TLR4 while increasing β-catenin (Ser552) and Akt (Ser473) phosphorylation in these cells. Knockdown of Foxo1 with siRNA administration in WT mice ameliorated lung injury and inhibited myeloperoxidase activity followed by rHMGB1 treatment, which was accompanied by decreased mRNA expression coding for TNF-α, IL-1β, MIP2, and IP-10. Moreover, Foxo1 knockdown inhibited the expression of TLR4-dependent IRF3 and IFN-β both in vitro and in vivo. These results demonstrate that PTEN/Foxo1 signaling is critical for triggering HMGB1-mediated innate TLR4 activation during ALI. By identifying the molecular signaling pathways within innate immune system, our studies provide the potential therapeutic targets for ALI.

## Introduction

Acute lung injury (ALI) and the acute respiratory distress syndrome (ARDS) are acute life-threatening disorders, which often results in multiorgan failure with a mortality of approximately 30–50 % [1, 2]. Currently, treatment options for ALI/ARDS are still limited to supportive measures and represent a major unmet clinical need, owing to lack of well understanding on host protective response to restrain acute inflammation in ALI/ARDS [1], implying the importance to elucidate the roles and mechanisms of inflammatory response in lung injury.

It has been known that activation of innate immune cells can trigger multiple intracellular signaling pathways during inflammatory response. The PI3K signaling plays an important role in regulating innate immunity, and its activation dampens the secretion of proinflammatory cytokines in myeloid cells [3]. These signaling processes are negatively regulated by PTEN, a lipid phosphatase, which acts as a tumor suppressor gene through the action of its phosphatase protein production [4]. Indeed, PTEN dephosphorylates PI(3,4,5)P3 to PI(4,5)P2, leading to antagonizing phosphoinositide 3-kinase (PI3K) [5, 6]. PTEN deficiency in mice leads to early embryonic lethality [7]. Enhanced PI3K/Akt activity by PTEN inhibition increases cardioprotection [8] and reduces brain damage [9]. Moreover, PTEN regulates LPS-induced TLR4 signaling and protects from endotoxic shock through a PI3K/Akt-dependent signaling [10]. Activation of PI3K/Akt negatively regulates NF-κB and the expression of inflammatory gene in macrophages [11], whereas deletion of PTEN in macrophages results in diminishing inflammation in response to TLR4 signaling [12]. Recently, myeloid PTEN activation promotes lung inflammatory response following bacterial infection [13], suggesting that macrophage PTEN plays a crucial role in regulating innate immunity in the lung injury.

Forkhead box proteins O (Foxos) are transcription factors, which were found to be critical to control cellular processes, including metabolism, cell differentiation, apoptosis, proliferation, and cellular stress resistance [14, 15]. The phosphorylation of Foxos by Akt can decrease its transactivation potential, leading to inhibiting DNA binding, nuclear exclusion, and subsequent sequestration into the cytoplasm [16]. However, dephosphorylation of Foxos increases its nuclear accumulation and activity, which in turn augments transcription of Foxo1 target gene expression [17]. Interestingly, Foxos have been shown to regulate innate immunity in lung infection [18]. Activation of Foxo reduces innate antimicrobial peptide (AMP), a host defense peptide, which is critical for the host innate immunity during inflammatory response [19]. Although these findings imply an essential role of Foxo in modulating cell processes and functions, little is known about the mechanistic links between PTEN and Foxo1 signaling in the regulation of immune homeostasis in ALI.

In the present study, we identify the novel role of PTEN/Foxo1 signaling in HMGB1-induced lung injury. HMGB1 induces PTEN, which in turn activates Foxo1 signaling, leading to inhibiting TLR4-driven inflammatory response. Furthermore, myeloid-specific PTEN knockout reduces Foxo1 activity and promotes β-catenin signaling, resulting in inhibiting TLR4-dependent signaling molecules to regulate lung inflammation. This study demonstrates that PTEN/Foxo1 signaling is critical for triggering HMGB1-mediated TLR4 activation in ALI.

## Materials and methods

### Animals

Male C57BL/6 wild-type (WT) mice at 8–10 weeks were purchased from The Jackson Laboratory (Bar Harbor, ME). The myeloid-specific PTEN knockout (PTEN^M-KO^) mice were generated as described [20]. All animals used were age- and sex-matched and housed in animal facility under specific pathogen-free conditions. The animals were fed a laboratory diet with water and food and kept under constant environmental conditions with 12 h light–dark cycles. All animal studies were approved by the institutional animal care and use committee at Anhui Medical University, Anhui, China.

### Mouse model and treatment

To establish the mouse model of acute lung injury (ALI), mice were anesthetized with i.p. ketamine (150 mg/kg) and acetylpromazine (13.5 mg/kg), and then, an incision (1–2 cm) was made on the animal neck to expose the trachea. Mice were instilled with recombinant HMGB1 (rHMGB1, Shino-TEST Co, Tokyo, Japan) (20 µg/mouse), diluted in 0.1 ml of sterile phosphate-buffered saline (PBS), via a 20-gauge catheter into the lumen of trachea, as described [21]. Control mice received the same volume of saline solution (8–10 mice per group). In some experiments, mice were injected i.v. with Foxo1 siRNA or non-specific (NS) siRNA (2 mg/kg, i.p.) (Santa Cruz Biotechnology, Inc.) at 4 h prior to rHMGB1 instillation, as described [22]. All animal studies were executed at 24 h after rHMGB1 treatment.

### Bronchoalveolar lavage fluid (BALF) and alveolar macrophage collection

The mice were anesthetized before exposure of the trachea. After the catheter was inserted into the lumen of trachea, the lungs were then lavaged three times with 0.8 ml of sterile saline. The total collected lavage averaged 1.4–1.7 ml/mouse. BALF was centrifuged at 800*g* for 8 min at 4 °C. The cell pellet was re-suspended in PBS and counted by a hemacytometer. The differential staining was performed with Diff-Quik staining solutions to count enriched alveolar macrophages as described [23]. The resulting cell consisted of >98 % macrophages, and cell viability was >95 %.

### Assessment of histology and myeloperoxidase activity

The lungs from mice were harvested and rinsed with PBS and then immersed into 10 % of buffered formalin overnight. After processing for paraffin embedding, the lung sections were stained with hematoxylin and eosin (H&E). The severity of lung injury was evaluated semiquantitatively by grading score on a scale from 1 to 5 as described [24]. The lung neutrophil accumulation was assessed by myeloperoxidase (MPO) activity assay [25]. One unit of MPO activity was defined as the quantity of enzyme degrading 1 μmol peroxide/min at 25 °C per gram of tissue.

### Western blot analysis

Protein was extracted from macrophages or lung tissues with ice-cold protein lysis buffer (50 mM Tris, 150 mM Nacl, 0.1 % sodium dodecyl sulfate, 1 % sodium deoxycholate, 1 % Triton-100). The buffer contains 1 % proteinase and phosphatase inhibitor cocktails (Sigma-Aldrich). Proteins (30 µg/sample) in SDS-loading buffer (50 mM Tris, pH 7.6, 10 % glycerol, 1 % SDS) were subjected to 4–20 % SDS–polyacrylamide gel electrophoresis (PAGE) and transferred to nitrocellulose membrane (Bio-Rad, Hercules, CA). The membrane was blocked with 5 % dry milk and 0.1 % Tween 20 (USB, Cleveland, OH). Monoclonal rabbit anti-mouse HMGB1, PTEN, phos-β-catenin (Ser552), β-catenin, phos-Akt (Ser473), Foxo1, TLR4, NF-κB and β-actin Abs (Cell Signaling Technology, MA) were used. The membranes were incubated with Abs and then developed according to the Pierce SuperSignal West Pico Chemiluminescent Substrate protocol (Pierce Biotechnology, Rockford, IL). Relative quantities of protein were determined and expressed in absorbance units (AU) comparing to β-actin expression using a densitometer (Kodak Digital Science 1D Analysis Software, Rochester, NY).

### Quantitative RT-PCR analysis

Total RNA was isolated from macrophages and lung tissues using RNAse Mini Kit (Qiagen, Valencia, CA) according to the manufacturer’s instructions. Reverse transcription to cDNA was performed by using SuperScript III First Strand Synthesis System (Invitrogen). Quantitative real-time PCR was performed using the DNA Engine with Chromo 4 Detector (MJ Research, Waltham, MA). In a final reaction volume of 25 μl, the following were added: 1× SuperMix (Platinum SYBR Green qPCR Kit; Invitrogen, San Diego, CA) cDNA and 10 μM of each primer. Amplification conditions were: 50 °C (2 min), 95 °C (5 min), followed by 40 cycles of 95 °C (15 s) and 60 °C (30 s). Primers used to amplify specific gene fragments were published [20]. Primer sequences for the amplification of TNF-α, IL-1β, MIP-2, CXCL-10, IRF3, IFN-β, and HPRT are shown: TNF-α forward, 5′-CTCCAGCTGGAAGACTCCTCCCAG-3′, reverse, 5′-CCCGACTACGTGCTCCTCACC-3′; IL-1β, forward, 5′-GCAACTGTTCCTGAACTCA-3′, reverse, 5′-CTCGGAGCCTGTAGTGCAG-3′; MIP-2 forward, 5′-GAACAAAGGCAAGGCTAACTGA-3′, reverse, 5′-AACATAACAACATCTGGGCAAT-3′; CXCL-10 forward, 5′-GCTGCCGTCATTTTCTGC-3′, reverse, 5′-TCTCACTGGCCCGTC ATC-3′; IRF3 forward, 5′-ACCAGCCGTGGACCAAGAG-3′, reverse, 5′-TACCAAGGCCCTGAGGCAC-3′; IFN-β, forward, 5′-CTCCTCCAAATTGCTCTCCTG-3′, reverse, 5′-GCAAACTGCTCACGAATTTTCC-3′; and HPRT forward, 5′- TCAACGGGGGACATAAAAGT-3′, reverse, 5′-TGCATTGTTTTACCAGTGTCAA’. These target gene expressions were calculated by their ratios to the housekeeping gene HPRT.

### In vitro cell culture and transfection

The alveolar macrophages were cultured in RPMI1640 medium (Invitrogen) supplemented with 10 % FBS, 100 μg/ml of penicillin/streptomycin (Life Technologies; Grand Island, NY). After 24 h cell culture, 1 × 10^6^ macrophages/well were transfected with 100 nM of Foxo1 siRNA using lipofectamine 2000 reagent (Invitrogen) and incubated for 24 h. Non-specific (NS) siRNA was used as a control. In some experiments, cells were pretreated with 10 µg/ml of rHMGB1 for 24 h.

### Malachite green phosphate assay

Murine alveolar macrophages protein lysates were immunoprecipitated with anti-PTEN Ab and incubated with protein A/G agarose beads. The PTEN malachite green assay was performed with beads-bound PTEN (Echelon Biosciences Inc., Salt Lake City, UT). The released phosphate was determined relative to a phosphatase standard curve.

### Statistical analysis

Data are expressed as mean ± SD and analyzed by permutation *t* test. Per comparison two-sided *p* values <0.05 were considered statistically significant. Multiple group comparisons were performed using one-way ANOVA with the post hoc test. All analyses were used by SAS/STAT software, version 9.4.

## Results

### HMGB1 induces ALI

**Fig. 1:**
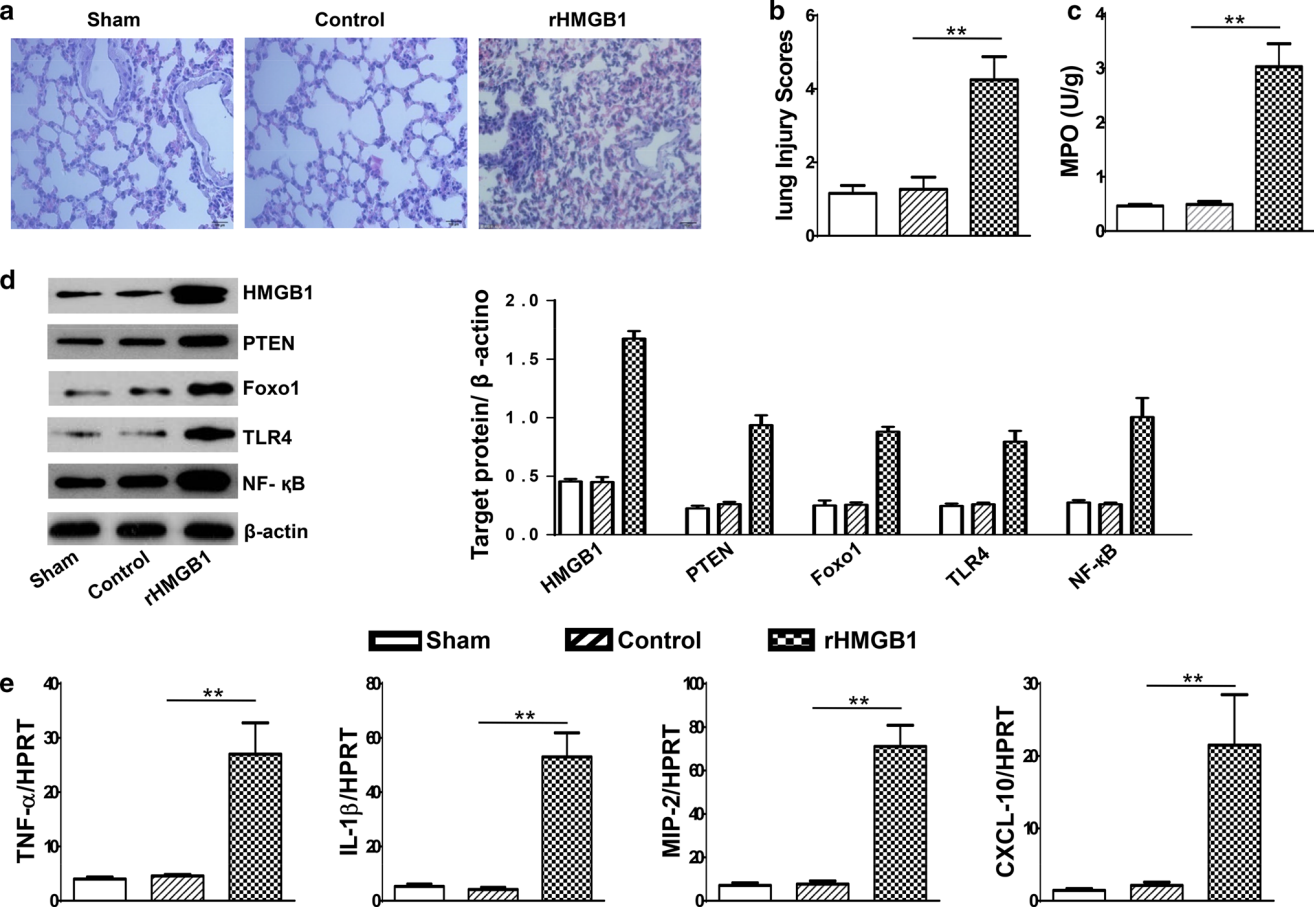
Activation of PTEN, Foxo1, and TLR4 in HMGB1-induced ALI. Wild-type (WT) mice were subjected to rHMGB1 instillation via a catheter after exposure of the trachea. **a** Lung sections were stained with H&E. Original magnification, ×40; **b** Histopathological mean lung injury scores (*n* = 8 animals per group); **p < 0.01. **c** MPO assay after rHMGB1 treatment (*n* = 8 samples/group), **p < 0.01. **d** The protein was isolated from lung tissues in sham, control, and rHMGB1-instilled mice. The expression of HMGB1, PTEN, Foxo1, TLR4, and NF-κB was analyzed by Western blots. Representative of three experiments. **e** q-PCR analysis of mRNA expression coding for TNF-α, IL-1β, MIP-2, and CXCL-10 in murine lungs after rHMGB1 treatment. Mean ± SD (*n* = 4–6 samples/group), **p < 0.01. Representive mean values of cytokine gene mRNA copies normalized to HPRT control. (*First bar*) sham, (*second bar*) control (PBS), and (*third bar*) rHMGB1

To examine whether HMGB1 contributes to ALI, WT mice were instilled with rHMGB1 into the lungs at the indicated doses, and lung histological evaluation was performed 24 h post-treatment. Mice treated with PBS control showed an almost normal lung structure. In contrast, rHMGB1 treatment displayed features of lung injury, including alveolar septal thickening, interstitial edema, vascular congestion, and neutrophil infiltration in the interstitium (Fig. 1a). In addition, the degree of lung injury was assessed by lung histopathology scores, which indicated severe lung injury in rHMGB1-treated mice, as compared with PBS-treated controls (Fig. 1b, 4.25 ± 0.63 vs. 1.27 ± 0.33, **p < 0.01). Moreover, lung MPO activity (U/g), an index of neutrophil accumulation, was increased in rHMGB1-treated mice, as compared with controls (Fig. 1c, 3.03 ± 0.42 vs. 0.49 ± 0.05; *p* < 0.01).

### HMGB1 increases PTEN and Foxo1 activity and activates innate TLR4 in ALI

To investigate whether HMGB1 plays a role in PTEN and Foxo1 activity in ALI, we performed Western blots to detect PTEN and Foxo1 genes (AU) in lung tissues after rHMGB1 instillation. Indeed, the expression of PTEN and Foxo1 was strongly upregulated in rHMGB1-treated lungs, as compared with PBS-treated controls (Fig. 1d). To further determine the role of TLR4 in initiating the inflammatory response in our experimental model, we detected the TLR4 expression in lungs after rHMGB1 treatment. Compared with PBS-treated controls, murine lungs instilled with rHMGB1 were characterized by increased activation of TLR4 and NF-κB (Fig. 1d), which was accompanied by increased mRNA levels coding for TNF-α, IL-1β, MIP-2, and CXCL-10 (Fig. 1e). Thus, these data suggest that HMGB1 induces innate immune responses through enhancing PTEN and Foxo1 activity in ALI.

### Myeloid-specific PTEN deficiency ameliorates lung damage in HMGB1-induced ALI

**Fig. 2:**
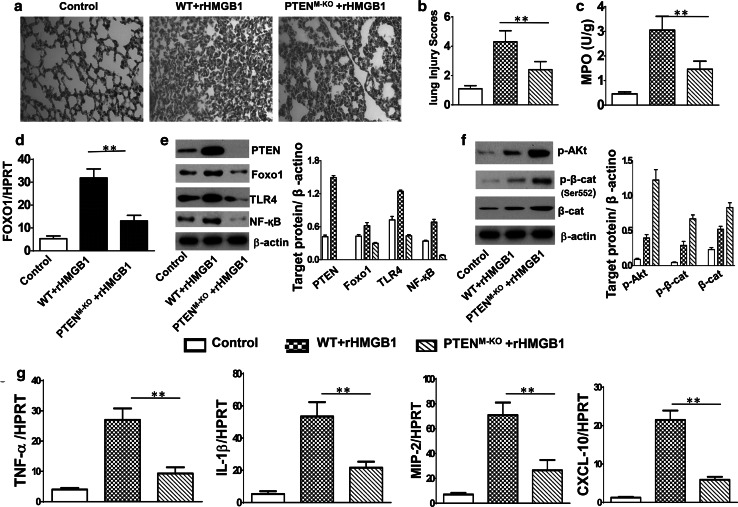
Myeloid-specific PTEN deficiency ameliorates lung damage and inhibits Foxo1 and TLR4 activation in HMGB1-induced ALI. WT and PTEN^M-KO^ mice were subjected to rHMGB1 instillation via a catheter after exposure of the trachea. **a** Lung sections were stained with H&E. Original magnification, ×40; **b** Histopathological mean lung injury scores (*n* = 8 animals per group); **p < 0.01. **c** MPO assay after rHMGB1 treatment (*n* = 8 samples/group), **p < 0.01. **d** q-PCR analysis of mRNA expression coding for Foxo1 in lungs from WT and PTEN^M-KO^ mice. Mean ± SD (*n* = 4–6 samples/group), **p < 0.01. **e** The protein was isolated from lung tissues in WT control, WT + rHMGB1, and PTEN^M-KO^ + rHMGB1 groups. The expression of PTEN, Foxo1, TLR4, and NF-κB was analyzed by Western blots. Representative of three experiments. **f** Western blot analysis of phos-Akt, phos-β-catenin(p-β-cat), and β-cat(β-cat). Representative of three experiments. **g** q-PCR analysis of mRNA expression coding for TNF-α, IL-1β, MIP-2, and CXCL-10 in lungs from WT and PTEN^M-KO^ mice after rHMGB1 treatment. Mean ± SD (*n* = 4–6 samples/group), **p < 0.01. Representive mean values of cytokine gene mRNA copies normalized to HPRT control. (*First bar*) WT control, (*second bar*) WT + rHMGB1, and (*third bar*) PTEN^M-KO^ + rHMGB1

As macrophages play an important role in orchestrating pulmonary innate immunity, we then asked whether myeloid-specific PTEN might affect HMGB1-mediated lung inflammation. In a mouse model of ALI, we found that deletion of PTEN expression in PTEN^M-KO^ mice reduced lung inflammatory response and its injury, evidenced by decreased lung interstitial congestion and inflammatory cell infiltration, as compared with WT followed by rHMGB1 treatment (Fig. 2a, b, 2.40 ± 0.55 vs. 4.31 ± 0.75, *p* < 0.01). Using MPO activity assay, we also found decreased lung neutrophil accumulation in PTEN^M-KO^ mice after HMGB1 stimulation, as compared with WT controls (Fig. 2c, 1.46 ± 0.33 vs. 3.05 ± 0.56, *p* < 0.01). These findings suggest that myeloid PTEN is an important mediator for the HMGB1-mediated inflammatory responses during the process of ALI.

### Myeloid-specific PTEN deficiency inhibits Foxo1 and TLR4 activation in HMGB1-induced ALI

We next investigated the role of PTEN in the regulation of Foxo1 and TLR4 in HMGB1-induced lung inflammation. Myeloid-specific PTEN knockout depressed lung expression of mRNA levels coding for Foxo1, compared with WT controls after rHMGB1 treatment (Fig. 2d, *p* < 0.01). Moreover, unlike enhanced HMGB1-induced Foxo1 and TLR4 activation seen in WT controls followed by rHMGB1 treatment, myeloid-specific PTEN ablation resulted in reduced Foxo1 and TLR4 expression, leading to the inhibition of NF-κB (Fig. 2e). Interestingly, PTEN deficiency in PTEN^M-KO^ but not in WT mice with rHMGB1 instillation augmented Akt (Ser473) and β-catenin (Ser552) phosphorylation (Fig. 2f), as well as decreased mRNA levels coding for TNF-α, IL-1β, MIP-2, and CXCL-10 (Fig. 2g). These findings suggest that PTEN is essential for the Foxo1 activation, and activation of β-catenin signaling might play an important role in the regulation of PTEN/Foxo1 signaling in HMGB1-induced ALI.

### Knockdown of Foxo1 signaling inhibits TLR4-driven inflammatory response in HMGB1-induced ALI

**Fig. 3:**
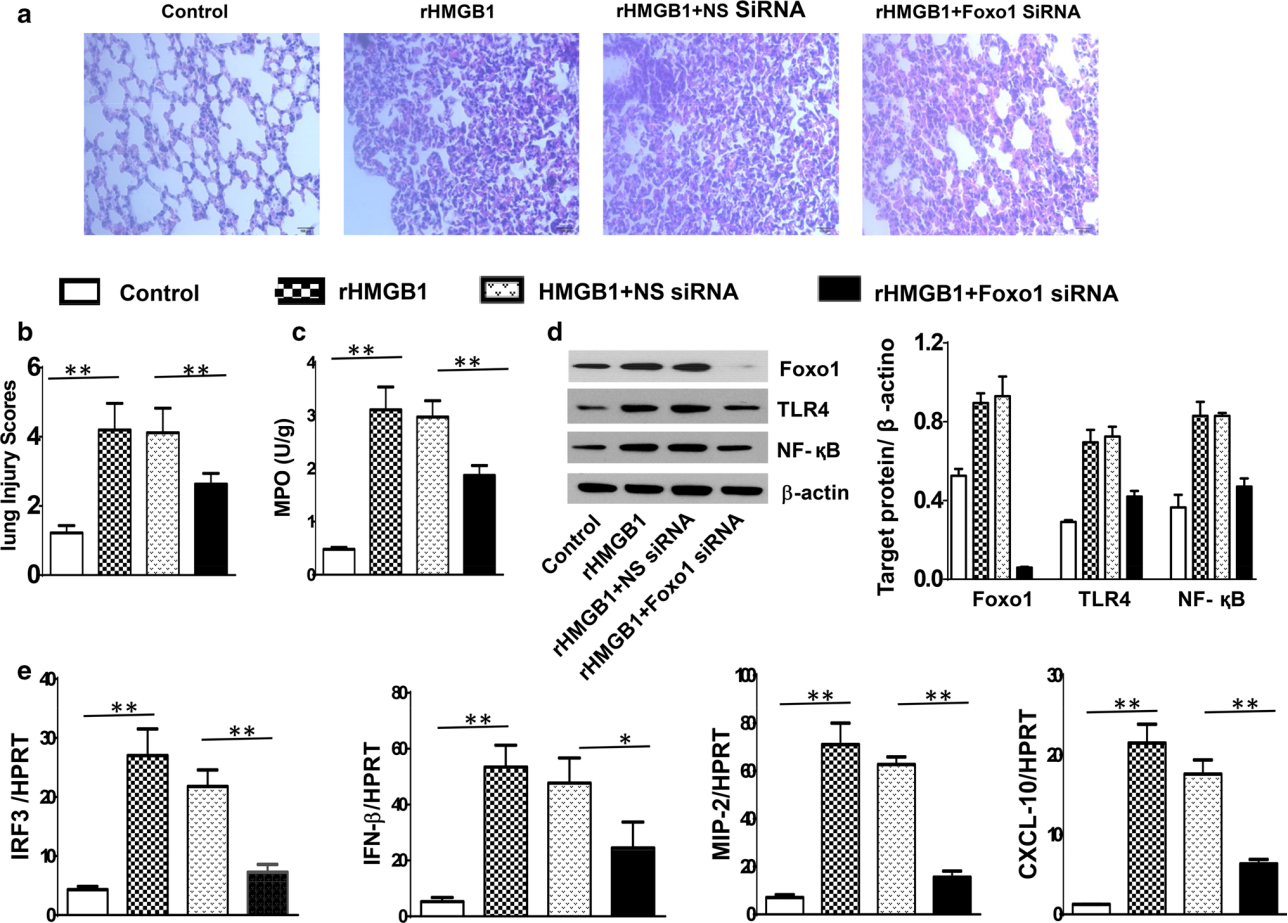
Knockdown of Foxo1 signaling inhibits TLR4-driven inflammatory response in HMGB1-induced ALI. WT mice were injected with Foxo1 siRNA or non-specific (NS) siRNA (2 mg/kg, i.p.) at 4 h prior to rHMGB1 instillation. **a** Lung sections were stained with H&E. Original magnification, ×40; **b** Histopathological mean lung injury scores (*n* = 8 animals per group); **p < 0.01. **c** MPO assay after rHMGB1 treatment (*n* = 8 samples/group), **p < 0.01. **d** The protein was isolated in lung tissues from control (PBS), rHMGB1, rHMGB1 + non-specific (NS) siRNA, and rHMGB1 + Foxo1 siRNA groups. The expression of Foxo1, TLR4, and NF-κB was analyzed by Western blots. Representative of three experiments. **e** q-PCR analysis of mRNA expression coding for IRF3, IFN-β, MIP-2, and CXCL-10 in lungs from WT mice after rHMGB1 treatment. Mean ± SD (*n* = 4–6 samples/group), *p < 0.05, **p < 0.01. Representive mean values of cytokine gene mRNA copies normalized to HPRT control. (*First bar*) control, (*second bar*) rHMGB1, and (*third bar*) rHMGB1 + NS siRNA (*fourth bar*) rHMGB1 + Foxo1 siRNA

To address the functional role of Foxo1 signaling in HMGB1-induced lung inflammation, we used siRNA that specifically targets Foxo1 in rHMGB1-treated mice. Interestingly, unlike non-specific (NS) siRNA-treated mice, which showed increased lung inflammatory response and neutrophil accumulation, knockdown of Foxo1 with siRNA administration did reveal less lung interstitial congestion and inflammatory cell infiltration (Fig. 3a, b, 4.12 ± 0.71 vs. 2.63 ± 0.31, *p* < 0.01), as well as decreased lung MPO activity (Fig. 3c, 1.87 ± 0.19 vs. 2.97 ± 0.31, *p* < 0.01). Moreover, NS siRNA treatment increased the expression of TLR4 and NF-κB followed by rHMGB1 instillation. In contrast, knockdown of Foxo1 resulted in inhibition of TLR4 and NF-κB (Fig. 3d). Consistent with these findings, the mRNA levels coding for IRF3, IFN-β, MIP-2, and CXCL-10 were consistently reduced in Foxo1 siRNA- but not in NS siRNA-treated mice (Fig. 3e).

### HMGB1 induces PTEN/Foxo1 signaling and triggers innate immune responses in vitro

**Fig. 4:**
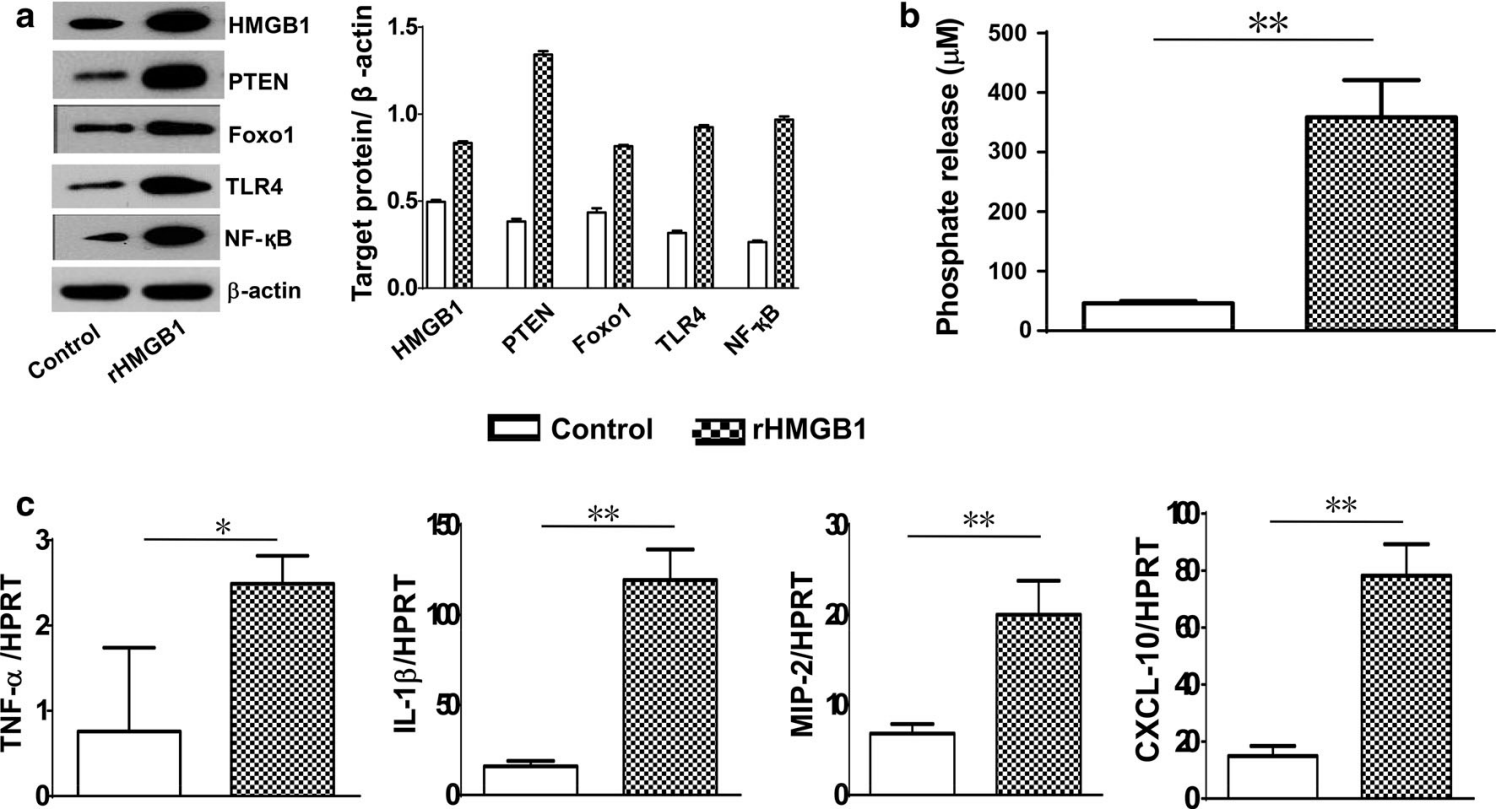
HMGB1 induces PTEN/Foxo1 signaling and triggers innate immune responses in vitro. The alveolar macrophages were isolated from WT mice and then incubated with rHMGB1 (10 µg/ml) for 24 h. **a** The protein was isolated from rHMGB1- or PBS-treated macrophages. The expression of HMGB1, PTEN, Foxo1, TLR4, and NF-κB was analyzed by Western blots. Representative of three experiments. **b** PTEN activity measured by malachite green phosphate assay. Mean ± SD; *n* = 4/group. **p < 0.01. **c** q-PCR analysis of mRNA expression coding for TNF-α, IL-1β, MIP-2, and CXCL-10 in alveolar macrophages after rHMGB1 treatment. Mean ± SD (*n* = 4–6 samples/group), *p < 0.05, **p < 0.01. Representive mean values of cytokine gene mRNA copies normalized to HPRT control. (*First bar*) control and (*second bar*) rHMGB1

Our in vivo data suggest that activation of both PTEN and Foxo1 signaling is important for the initiation of inflammatory response in HMGB1-induced ALI. To further elucidate the cross talk between PTEN and Foxo1 in HMGB1-mediated lung inflammation, mouse alveolar macrophages were cultured and stimulated with rHMGB1. Unlike in PBS-treated control cells, Western blot analysis showed that rHMGB1 treatment markedly augmented PTEN and Foxo1, leading to enhanced TLR4 and NF-κB activation in alveolar macrophages (Fig. 4a). Moreover, we used PTEN phosphate release assay, in which rHMGB1 treatment resulted in increased PTEN activity (Fig. 4b), as compared with control cells. These data were consistent with increased mRNA levels for TNF-α, IL-1β, MIP-2, and CXCL-10 in rHMGB1-treated cells (Fig. 4c). Hence, HMGB1 activates PTEN/Foxo1 signaling and mediates TLR4-driven inflammatory response.

### Macrophage PTEN deficiency inhibits Foxo1 activity and activates β-catenin signaling in HMGB1-mediated inflammation in vitro

**Fig. 5:**
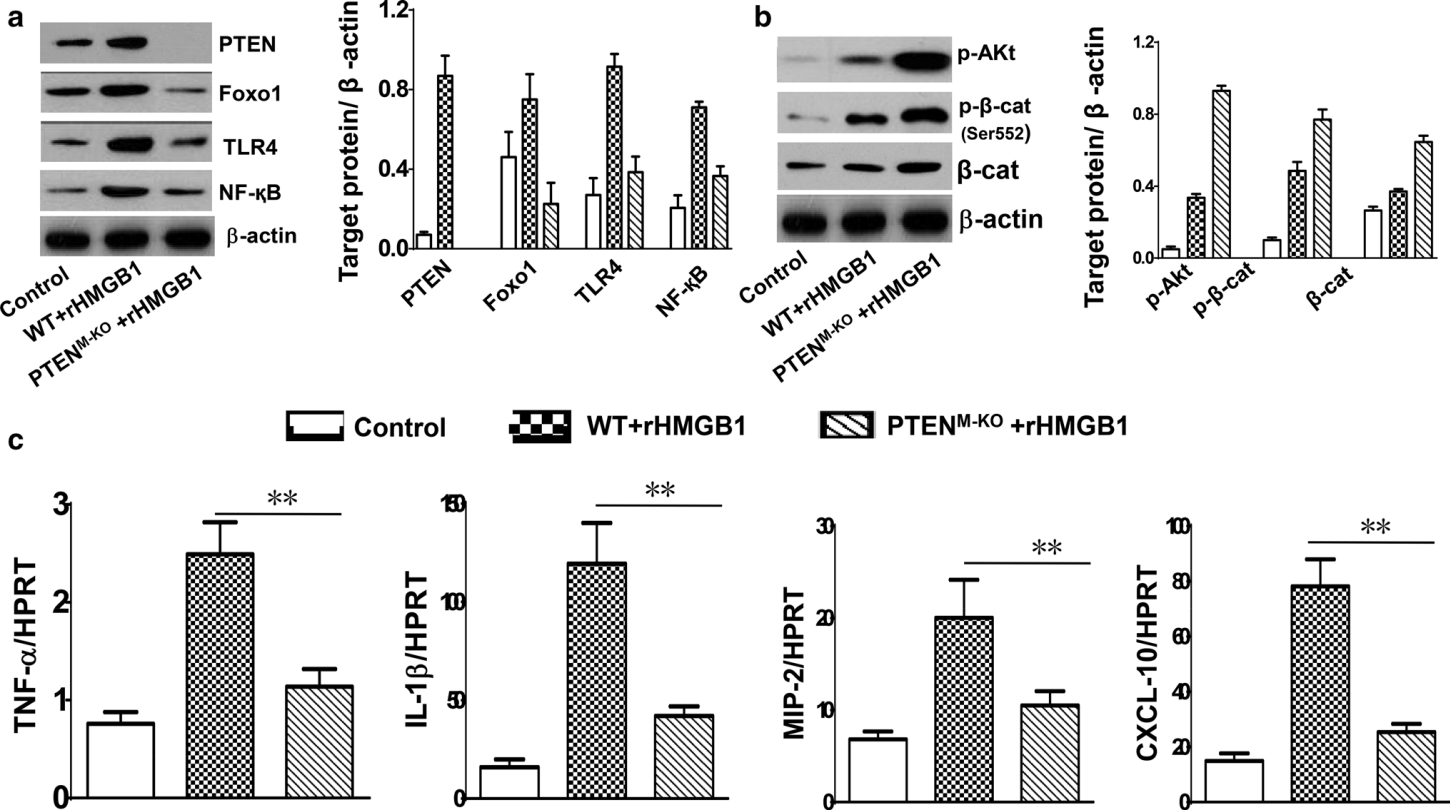
Macrophage PTEN deficiency inhibits Foxo1 activity and activates β-catenin signaling in HMGB1-mediated inflammation in vitro. The alveolar macrophages were isolated from WT and PTEN^M-KO^ mice and then incubated with rHMGB1 (10 µg/ml) for 24 h. **a** The protein was isolated from rHMGB1-treated macrophages. The expression of PTEN, Foxo1, TLR4, and NF-κB was analyzed by Western blots. Representative of three experiments. **b** Western blot analysis of phos-Akt, phos-β-catenin(p-β-cat), and β-catenin(β-cat). Representative of three experiments. **c** q-PCR analysis of mRNA expression coding for TNF-α, IL-1β, MIP-2, and CXCL-10 in alveolar macrophages after rHMGB1 treatment. Mean ± SD (*n* = 4–6 samples/group), **p < 0.01. Representive mean values of cytokine gene mRNA copies normalized to HPRT control. (*First bar*) WT control, (*second bar*) WT + rHMGB1, and (*third bar*) PTEN^M-KO^ + rHMGB1

To delineate the importance of PTEN/Foxo1 signaling in HMGB1-mediated inflammatory response in our experimental model, we cultured alveolar macrophages from PTEN^M-KO^ mice. Compared with macrophages from PTEN proficient (WT) counterparts, cells from PTEN^M-KO^ mice were characterized by decreased Foxo1, TLR4, and NF-κB (Fig. 5a) yet increased Akt (Ser473) and β-catenin (Ser552) activation (Fig. 5b), which was accompanied by decreased mRNA expression coding for TNF-α, IL-1β, MIP-2, and CXCL-10 in rHMGB1-treated cells (Fig. 5c). Thus, macrophage PTEN deficiency inhibits Foxo1 activity and activates β-catenin signaling, leading to reduced TLR4/NF-κB activation in HMGB-mediated lung inflammation.

### Disruption of Foxo1 signaling regulates TLR4-dependent gene programs in HMGB1-mediated inflammation in vitro

**Fig. 6:**
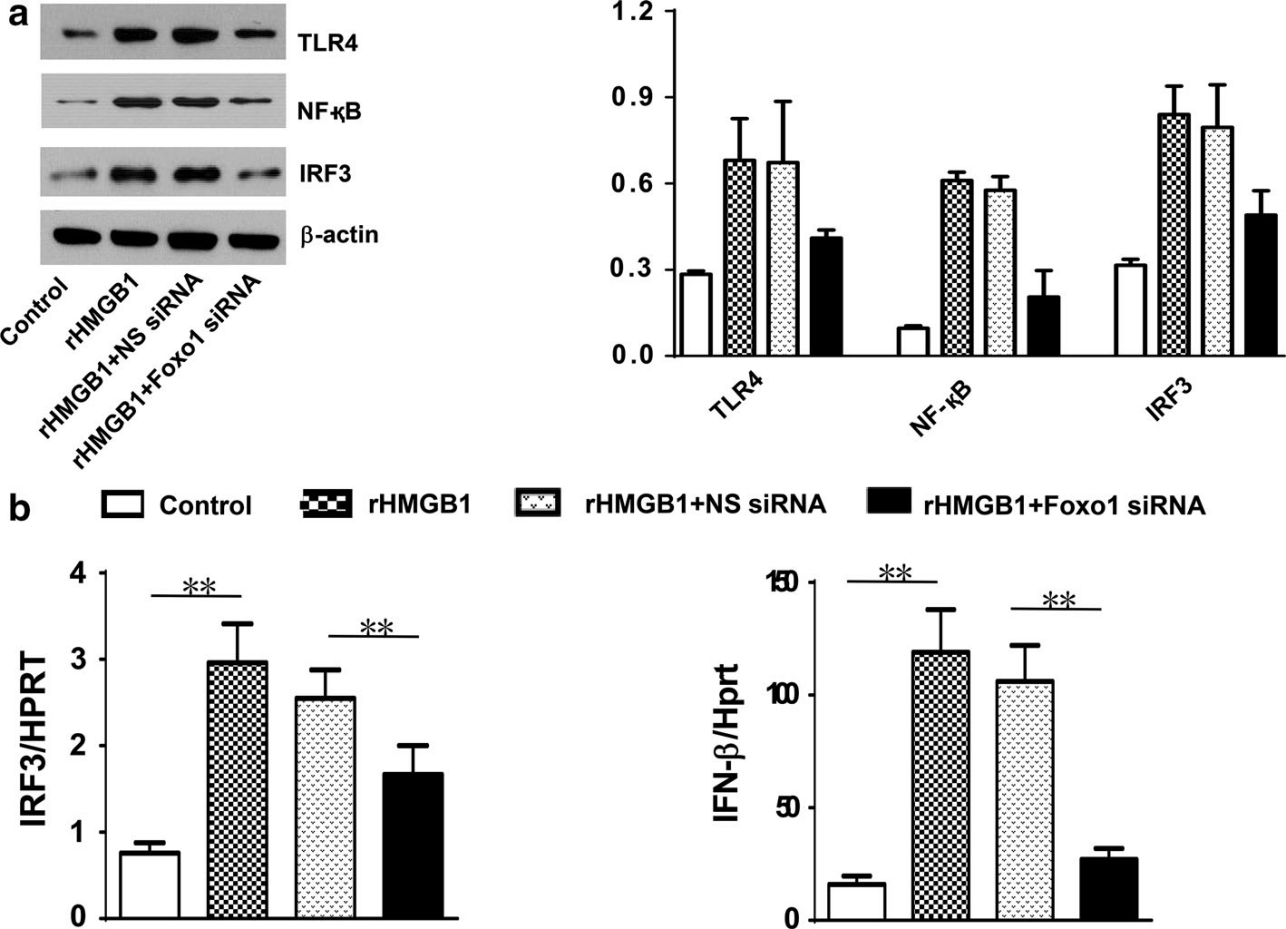
Disruption of Foxo1 signaling regulates TLR4-dependent gene programs in HMGB1-mediated inflammation in vitro. The alveolar macrophages were isolated from WT mice and then transfected them with Foxo1 siRNA or non-specific (NS) siRNA (100 nM). The cells were cultured for 24 h. **a** The protein was isolated from Foxo1 siRNA- or NS siRNA-transfected macrophages. The expression of TLR4, NF-κB, and IRF3 was analyzed by Western blots. Representative of three experiments. **b** q-PCR analysis of mRNA expression coding for IRF3 and IFN-β in Foxo1 siRNA- or NS siRNA-transfected macrophages. Mean ± SD (*n* = 4–6 samples/group), **p < 0.01. Representive mean values of cytokine gene mRNA copies normalized to HPRT control. (*First bar*) control (PBS), (*second bar*) rHMGB1, and (*third bar*) rHMGB1 + NS siRNA (*fourth bar*) rHMGB1 + Foxo1 siRNA

As disruption of PTEN-mediated Foxo1 signaling contributed to the inhibition of TLR4/NF-κB activity, we next asked whether PTEN-mediated Foxo1 might affect TLR4-specific targeting gene programs in macrophages. We cultured alveolar macrophages from WT mice and then transfected these cells with Foxo1 siRNA following rHMGB1 treatment. In contrast to NS siRNA-treated cells, knockdown of Foxo1 with siRNA treatment reduced IRF3 protein expression after rHMGB1 treatment (Fig. 6a). Consistent with these findings, decreased mRNA levels coding for IRF3 and IFN-β were observed in Foxo1 siRNA-treated cells, as compared with NS siRNA-treated controls (Fig. 6b). These results suggest that PTEN/Foxo1 signaling mediates TLR4-specific signals, resulting in a gene expression profile.

## Discussion

This study documents the essential role of PTEN/Foxo1 signaling in innate immune responses that orchestrate TLR4-driven lung inflammation in HMGB1-induced ALI. First, HMGB1 induces PTEN and Foxo1 activation to trigger innate TLR4-mediated inflammatory response. Second, myeloid-specific PTEN knockout reduces Foxo1 activity and promotes β-catenin signaling, leading to inhibiting TLR4/NF-κB activation and decreasing proinflammatory cytokine and chemokine mediators. Third, disruption of Foxo1 signaling depresses TLR4-mediated signaling molecules to regulate lung inflammatory response. Our results delineate the roles of PTEN/Foxo1 signaling in triggering innate TLR4-driven inflammatory response during HMGB1-induced ALI. We also demonstrate that disruption of PTEN/Foxo1 signaling contributes to the inhibition of lung inflammation. Our study supports a molecular mechanism by which disruption of PTEN/Foxo1 signaling regulates TLR4-mediated innate immunity, a novel approach for the management of innate immunity-driven lung injury.

The molecular mechanism of HMGB1-induced lung injury involves activation of multiple signaling pathways [26]. PTEN, a multifunctional phosphatase, was shown to be essential in lung injury through regulation of its downstream PI3K/Akt signaling [5]. PTEN negatively regulates PI3K/Akt pathway by metabolizing phosphatidylinositol 3,4,5-trisphosphate [PtdIns(3,4,5)P(3)] and acts in direct antagonism to PI3-kinases, leading to inactivation of Akt [27]. Indeed, Akt is a key signaling protein in the cell survival pathways [28]. Activation of Akt increases cell survival by phosphorylating and inhibiting Foxo1, leading to reducing cell apoptosis [14]. Consistent with the role of PI3K/Akt signaling cascade in the innate immune response [3], our current in vivo study has shown that activation of PTEN increased HMGB1-induced lung inflammatory injury. However, myeloid-specific PTEN deficiency diminished the inflammatory response, evidenced by ameliorated lung damage, reduced macrophage and neutrophil activation, as well as proinflammatory cytokine/chemokine gene expression. More importantly, myeloid-specific PTEN deficiency resulted in inhibition of Foxo1 but activation of Akt and β-catenin, which implies the specific interactions between PTEN/Foxo1 signaling and β-catenin activation in the regulation of HMGB1-mediated lung inflammation.

Our in vitro and in vivo data showed that activation of PTEN increased Foxo1 activity, whereas myeloid-specific PTEN deficiency increased Akt phosphorylation, which in turn phosphorylated Foxo1, resulting in increasing its exportation from the nucleus to the cytoplasm and reducing its DNA-binding capacity, thereby inhibiting Foxo1 transcriptional activity. Consistent with the Foxo1 functions in regulating innate immunity during inflammatory response [19], we found that disruption of Foxo1 signaling decreased TLR4-mediated IRF3 and IFN-β expression in HMGB1-mediated lung inflammation. Indeed, IRF3 plays an important role in the innate immune response to viral infection [29]. IRF3 can be activated by TLR4-dependent signaling [30]. In concert with NF-κB, IRF3 transactivates the IFN-β gene [31], as well as IRF3-dependent genes CXCL-10, and CCL5 [32, 33], suggesting that a direct interaction between Foxo1 signaling and TLR4-mediated signaling molecules triggers innate immune response in lung injury.

To further elucidate the mechanism by which PTEN/Foxo1 signaling may regulate HMGB1-induced lung inflammation, we isolated alveolar macrophages in BALF from rHMGB1-instilled lungs. Indeed, activation of alveolar macrophages is a key for triggering innate TLR4-mediated inflammatory responses in the development of ALI [34]. rHMGB1 treatment in alveolar macrophages increased PTEN and Foxo1 activity. However, macrophage PTEN deficiency increased Akt and β-catenin (Ser552) phosphorylation, which resulted in increased translocation of β-catenin into the nucleus, enhancing its transcriptional activity [35]. These are consistent with the report of activated PI3K/Akt to promote β-catenin signaling in cardiomyocytes [36]. Indeed, the β-catenin has been shown the ability to limit inflammatory responses by controlling DC function and inducing anti-inflammatory mediators [37, 38]. Activation of β-catenin inhibits NF-κB by impairing its DNA binding/transcription coding activity, leading to depressed expression of NF-κB target genes, including proinflammatory cytokine and chemokine mediators [39]. These results were further supported by our in vivo data, which myeloid-specific PTEN knockout increased Akt and β-catenin phosphorylation but decreased nuclear Foxo1 activity, leading to inhibition of TLR4-mediated lung inflammation even though rHMGB1 treatment. Therefore, our results provide direct evidence that PTEN/Foxo1 signaling triggers innate TLR4-driven inflammatory through an Akt/β-catenin-dependent signaling pathway in HMGB1-induced lung injury.

In conclusion, our findings suggest that PTEN/Foxo1 signaling is critical for triggering HMGB1-mediated innate TLR4 activation in ALI. Myeloid-specific PTEN ablation activates β-catenin, which then inhibits Foxo1, leading to regulation of TLR4-driven inflammatory response. By identifying molecular mechanisms of PTEN/Foxo1 signaling in TLR4-mediated innate immunity, our study provides the rationale for novel therapeutic approaches that can be applied to future translational and clinical studies in ALI.
